# An Improved Racetrack Structure for Transporting a Skyrmion

**DOI:** 10.1038/srep45330

**Published:** 2017-03-30

**Authors:** P. Lai, G. P. Zhao, H. Tang, N. Ran, S. Q. Wu, J. Xia, X. Zhang, Y. Zhou

**Affiliations:** 1 College of Physics and Electronic Engineering, Sichuan Normal University, Chengdu 610101, China; 2 Department of Physics and Electronic science, Aba Teachers University, Wenchuan 623002, China; 3 Collaborative Innovation Center for Shanxi Advanced Permanent Materials and Technology, Linfen 041004, China; 4 School of Science and Engineering, The Chinese University of Hong Kong, Shenzhen 518172, China

## Abstract

Magnetic skyrmions are promising building blocks for next generation data storage due to their stability, small size and extremely low currents to drive them, which can be used instead of traditional magnetic domain walls to store information as data bits in metalic racetrack memories. However, skyrmions can drift from the direction of electron flow due to the Magnus force and thus may annihilate at the racetrack edges, resulting in the loss of information. Here we propose a new skyrmion-based racetrack structure by adding high-*K* materials (materials with high magnetic crystalline anisotropy) at the edges, which confines the skyrmions in the center region of the metalic racetrack efficiently. This design can overcome both the clogging and annihilation of skyrmions according to our micromagnetic simulation, which occur normally for skyrmions moving on a racetrack under small and large driving currents, respectively. Phase diagrams for skyrmion motion on the proposed racetrack with various values of current density and racetrack edge width have been calculated and given, showing that skyrmions can be driven at a high speed (about 300 m/s) in the racetrack under relatively smaller driving currents. This design offers the possiblity of building an ultrafast and energy-efficient skyrmion transport device.

The magnetic skyrmion is a topologically stable magnetization configuration, as the potential information carrier unit in the future, which has attracted widely research interests due to their small size, stability and the extremly low current needed to move them^1^,^2^,^3^,^4^,^5^,^6^,^7^,^8^,^9^,^10^,^11^,^12^,^13^,^14^,^15^. The skyrmionic state is stable in confined films, in addition to the domain wall state and the cycloidal state, which can be adjusted by material and geometric parameters^16^. Skyrmion-based racetrack memory^17^,^18^,^19^ is expected to have improved storage density and lower energy consumption in comparison with the racetrack memory based on domain walls^20^,^21^,^22^. Many progresses have been made on the dynamics of current-induced skyrmion motion^2^,^3^,^8^,^14^,^23^, however, there is still a long way for putting the skyrmion-based racetrack memory to practice. For example, to create, drive and annihilate/destroy the skyrmions or the skyrmion cluster^24^,^25^ at will is still a challenge. In particular, current-driven skyrmions will drift from the racetrack direction due to the presence of Magnus force^14^,^20^,^26^ if the velocity is high enough, which leads to their annihilation at the racetrack edge and the loss of information.

Various potential barriers have been proposed to confine skyrmions in the center region of the racetrack so that the annihilation at the racetrack edge is avoided^27^,^28^,^29^,^30^,^31^. For example, the potential barriers can be created by tuning the perpendicular magnetic anisotropy (PMA)^30^, the height of the ferromagnetic layers or the damping constant in the racetrack edges^29^,^31^. The PMA can be tuned by accurately controlled light ion irradiation so that the lower magnetic anisotropy at the center can be formed in contrast to that at the edges. As a result, a path of lower resistance is created at the racetrack center, allowing the skyrmions to pass the racetrack without annihilation. The height of the ferromagnetic layers can be tuned by creating a rectangular groove on the center of the racetrack. A curb structure is thus formed, which functions to confine the skyrmion within the groove. The damping constant of the racetrack can be tuned in either the transverse or the longitudinal direction to avoid the annihilation of the skyrmion. In either case, the deviations of the skyrmions can be in opposite directions in different regions of the racetrack if the damping constants are properly tuned, which cancel each other so that the skyrmion can be efficiently confined in the racetrack center. All these methods are similar in that some effective energy barriers are created, which confine the skyrmion within the center of the racetrack. However, the method to create the energy barrier is quite different. The PMA is tuned by light ion irradiation while the curb structure is created geometrically. On the other hand, the skyrmion is confined near the racetrack center due to the cancellation of the opposite drift velocities in different regions of the racetrack if the damping constant is properly modulated.

In this work, we propose a simple new method to avoid the skyrmion annihilation at a high speed by adding a material with high crystalline anisotropy^22^ at the two symmetrical edges of a 0.4-nm-thick and 400-nm-long perpendicularly magnetized racetrack, as shown in Fig. 1. Our micromagnetic simulation demonstrates that the skyrmion can move in the racetrack without clogging under a small driving current. In particular, the driving current needed is much smaller than other methods^2^,^18^,^29^,^30^ to achieve the same skyrmion velocity.

## Results and Discussions

### The motion and oscillation of a skyrmion in the racetrack

**Figure 2:**
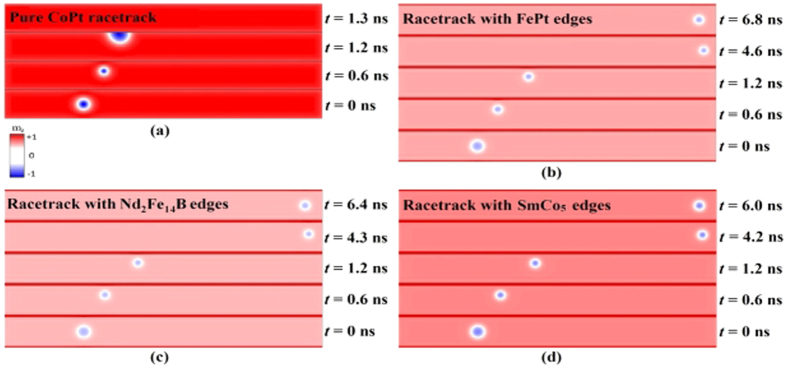
Snapshots of simulation at various times *t* for a skyrmion driven by the spin current of 6 MA/cm^2^ in a racetrack made of a single material of CoPt with a width of 40 nm and with a high-*K* material rimmed at the edges. In (**b**), (**c**) and (**d**), FePt, Nd_2_Fe_14_B and SmCo_5_ are used as edge materials respectively, with the width *w*_*edge*_ equal to 2 nm. The out-of-plane component of the magnetization is indicated by the color scale, which has been used throughout this paper.

Figure 2 shows the snapshots of skyrmion motion on various racetracks at different simulation times. For a racetrack with only one material of CoPt as shown in Fig. 2(a), the skyrmion can stay at the racetrack for only about 1.2 ns, which moves in the *x* direction driven by the injected current of 6 MA/cm^2^. At *t* = 1.2 ns, the skyrmion touches the upper edge of the racetrack due to the Magnus force and loses its topological stable skyrmion state, which annihilates eventually within 0.1 ns so that there is no skyrmion left in the racetrack at *t* = 1.3 ns.

To avoid the undesired annihilation of skyrmions during the motion, we have set the racetrack with various high-*K* materials at the upper and lower edges as shown in Fig. 2(b–d), where skyrmions can be kept in the racetrack for the whole simulation period. In this case, skyrmions will still drift towards the upper edge of the racetrack initially due to the Magnus force for *t* < 1.2 ns, similar to the case with only one material shown in Fig. 2(a). However, when the skyrmion is close to the upper edge of the racetrack (*t *=* *1.2 ns), it feels a large repulsion force from the upper edge due to the frame of the high-*K* material there. This repulsion force is larger than the Magnus force when the skyrmion is very close to the upper edge so that it drifts back to the center of the racetrack. Once the skyrmion is away from the upper edge, the repulsion force becomes much smaller so that the oscillation for the displacement of the skyrmion in the *y* direction will occur. Such an oscillation will continue until the skyrmion reaches the right end of the racetrack, which occurs at *t* = 4.6 ns for a racetrack set with a FePt edge shown in Fig. 2(b).

After that, it bounces back and oscillates in the horizontal direction due to the repulsion from the right end of the racetrack while keeping in the center axis of the racetrack. The oscillation will continue for a few nanoseconds and finally the skyrmion stops still at the racetrack. It is worth mentioning that the oscillation of a moving skyrmion due to the skyrmion-edge repulsion effect has been observed in a recent experiment work^32^,^33^.

Such a skyrmion-edge effect at the end of the racetrack has been systematically investigated in ref. 18, which will cause the clogging of the skyrmions if a skyrmion chain is driven by the current in the racetrack. The clogging can be avoided by increasing the diving current^3^, or rather, by adding a notch at the end of the racetrack^18^. The later method could be more energy efficient.

One can see that the motions of the skyrmions are not sensitive to the particular material selected to rim the edges of the racetrack, where the competition between the STT effect^34^ and the boundary confinement determines the moving behaviors of skyrmion. As a result, in Fig. 2(b–d), all three skyrmions can pass to the right end of the racetrack successfully and finally stop still there.

### The optimization of the skyrmion racetrack

**Figure 3:**
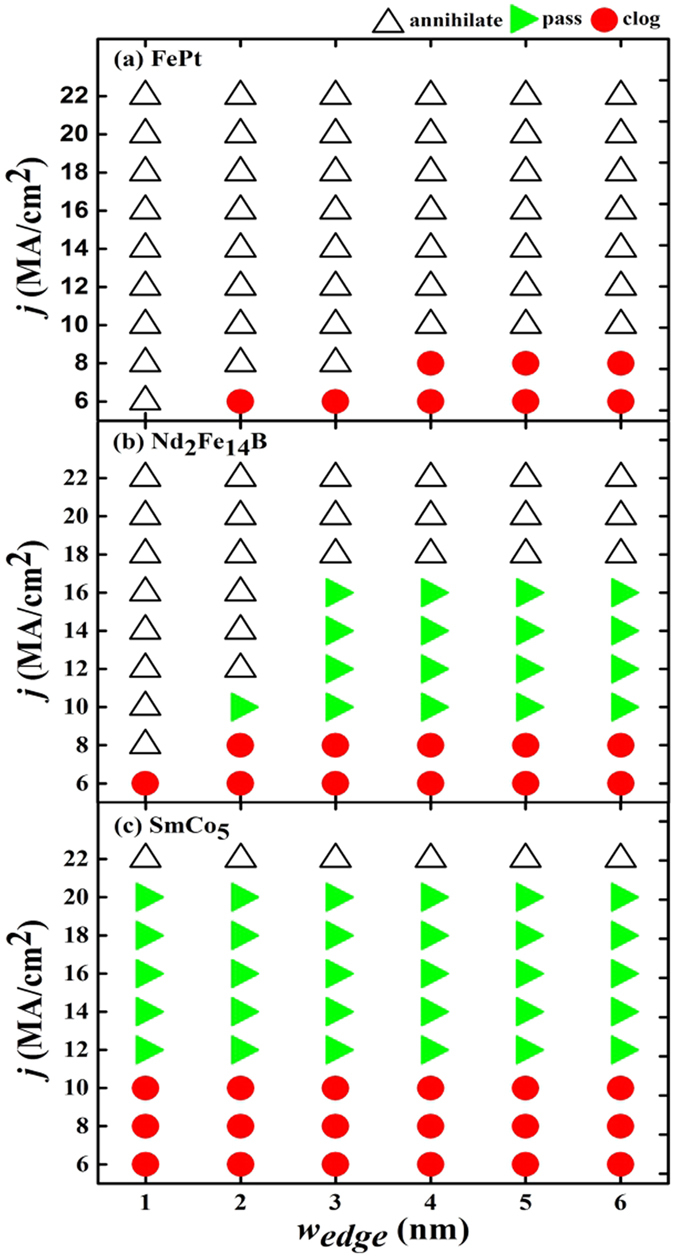
Calculated phase diagrams for the motion of the skyrmions with various values of the racetrack edge width and the current density. The open triangles denote the phase where skyrmions will annihilate by touching the upper edge due to the Magnus force. The filled green triangle corresponds to the skyrmion phase at which it can reach the right end of the racetrack and pass through it. The red circle stands for the phase where skyrmions will clog at the right end of the racetrack as shown in Fig. 2(b–d).

The simplest way to avoid clogging is to increase the current density. Figure 3 shows the calculated phase diagrams for skyrmion motion on the racetrack with various values of current density and racetrack edge width. From Fig. 3(c), it can be seen that for the current density (*j* = 6, 8, 10 MA/cm^2^), the skyrmion moving on the racetrack are ultimately clogged at the right end due to the repulsion from the right end of the racetrack if the upper edge is rimmed with SmCo_5_, which has a very high crystalline anisotropy. Increase of the driving current density will lead to successful passing of the skyrmions through the right end of the racetrack. It should be noted, however, the enhancement of the current density will increase the Magnus force and hence the drift velocity toward the upper edge. As a result, the skyrmions will touch the upper edge and annihilate there when the current density increases to 22 MA/cm^2^ as shown in Fig. 3(c). In contrast, the skyrmions will either be clogged at the right end (with a small current) or be destroyed at the upper edge (with a large current) for the racetrack with FePt edges, where the crystalline anisotropy of the edge material is not large enough. Similar clogging and annihilation can be observed for a racetrack with pure CoPt, which are not shown here. If the edge material is Nd_2_Fe_14_B (which has a crystalline anisotropy in between), on the other hand, the skyrmions will pass through the right end successfully in most cases as shown in Fig. 3(b). Annihilation of the skyrmions at the upper edge can take place only when the current density is very large (*j* ≥ 18 MA/cm^2^) or when the edge material is very thin (*w*_*edge*_ = 1 nm or 2 nm).

**Figure 4:**
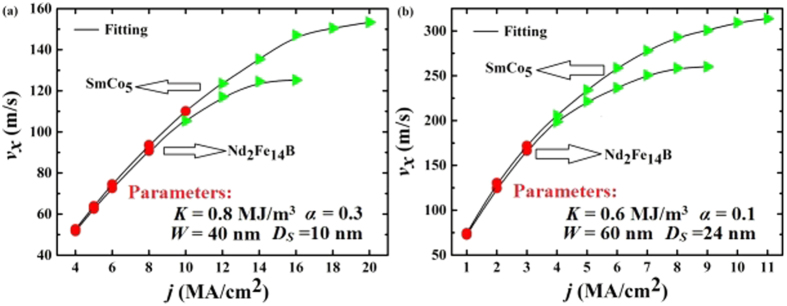
Calculated stable velocities *v*_*x*_ of the skyrmions along the racetrack direction as functions of the current density with two different sets of parameters for the racetrack designed. The red and green filled symbols denote the skyrmions clogging at the right end of the racetrack and passing the right end of the racetrack, respectively. The solid lines in Fig. 4(b) show two fitted lines for the edged racetracks respectively. Both racetracks in Fig. 4(a) and (b) are edged with 6-nm-wide high-*K* materials with the same spin polarization coefficient (*p* = 0.4). The different parameters used in Fig. 4(a) and (b) are indicated in the figures, which are the magneto-crystalline anisotropy constant *K*, damping constant *α*, the inner CoPt racetrack width *W* and the diameter of the skyrmion *D*_*S*_, respectively.

From the above discussions, it is clear that Nd_2_Fe_14_B and SmCo_5_ are preferred edge materials to be used. Therefore, we have calculated the velocity as functions of the driving current for various racetracks rimmed with 6 nm Nd_2_Fe_14_B and SmCo_5_ as shown in Fig. 4.

One can see from Fig. 4 that all the velocities increase with the injected current parabolically and the skyrmions can pass through the right end successfully in a wide range of driving current. For larger driving currents, the skyrmions will annihilate at the upper edges, whereas they will clog at the right end at a smaller driving current.

Figure 4(a) shows the current dependent velocity with the same parameters as Figs 2 and 3, which are adopted from ref. 2. The efficiency for the velocity versus the current is about 10 m/s per MA/cm^2^, agreeing well with that calculated by other groups for pure CoPt racetrack with a vertical current injection^2^. The important point, however, is that the present design overcomes the clogging and annihilation problems for a pure CoPt racetrack. To enhance the skyrmion speed, we have improved the racetrack design by adjusting the parameters, which are demonstrated in Fig. 4(b).

The skyrmion speed in Fig. 4(b) can reach 300 m/s, which is much larger than those for other skyrmion racetrack designed, as summarized in Table 1. In particular, the driving current in the present work is much smaller than those used by ref. 29, where a curb was utilized in the CoPt racetrack to prevent the skyrmions from annihilation. In addition, the polarization adopted in this calculation is 0.4, which is much easier to be realized in experiments than that used in ref. 29, where a much larger polarization of 0.7 was used. The efficiency for the velocity versus the current in this work is about 40 m/s per MA/cm^2^, also much larger than that calculated by other groups^2^,^8^,^18^,^29^,^30^,^35^. It is noted that the skyrmion size and track width in Fig. 4(b) are larger than those in Fig. 4(a) and those by other groups whilst the crystalline anisotropy *K* for CoPt is smaller, as shown in Table 2, which can increase the skyrmion speed in a racetrack significantly according to Ding *et. al*.^35^. The smaller damping constant *α* adopted in Fig. 4(b) is an additional factor that enhances the skyrmion speed according to Thiele equation^36^. Overall, racetrack designed in Fig. 4(b) is much better than that in Fig. 4(a), demonstrating a much larger skyrmion speed whilst the driving current needed is much smaller.

The relation between the skyrmion speed and the driving current in Fig. 4(b) can be well fitted by two parabolic functions, which are 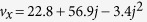 and 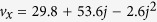 for Nd_2_Fe_14_B and SmCo_5_ respectively. For small driving current, the skyrmion speed is roughly proportional to the driving current, consistent with those observed in other literatures^2^,^8^,^18^,^35^. When the current is larger, however, the skyrmion speed becomes saturated. Therefore, the optimum current injected is 4–10 MA/cm^2^ with Nd_2_Fe_14_B or SmCo_5_ as edge materials.

Further, our calculation shows that the skyrmion velocity is not sensitive to the DMI at the edges and the much smaller DMI there are not the key for the hinder of annihilation, as illustrated in Fig. S1 for a CoPt racetrack rimmed with Nd_2_Fe_14_B edges.

### Time evolution of the skyrmion energy and the lateral displacement

**Figure 5:**
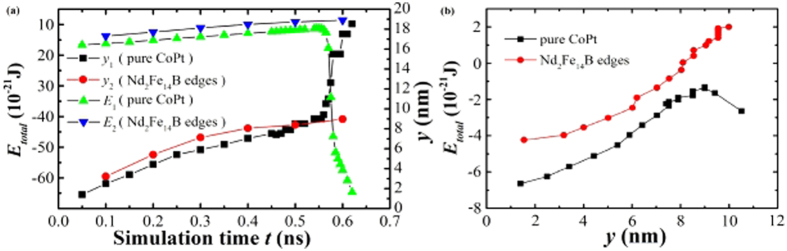
(**a**) Comparison of calculated total energy and lateral displacement *y* as functions of the simulation time for skyrmions driven on a CoPt racetrack rimed with 6 nm Nd_2_Fe_14_B edges and a pure 40 nm CoPt racetrack respectively. (**b**) Calculated total energy as the function of the lateral displacement *y* for two types of racetrack, demonstrating that the large energy gradient at the racetrack edge due to high-*K* Nd_2_Fe_14_B which prevents the sharp lateral displacement and hence the annihilation of the skyrmion. The driven current for the skyrmion in each case is 8 MA/cm^2^, whilst the initial skyrmion energy (*t* = 0) and the racetrack center are set as zero points for the total energy and lateral displacement, respectively.

To understand why the high-*K* edges can confine the skyrmions within the racetrack, the evolution of the total energy has been calculated for a CoPt racetrack with Nd_2_Fe_14_B edges and a pure CoPt, shown in Fig. 5(a). For *t* < 0.55 ns, the total energies of the skyrmions in both racetracks increase linearly with *t*, accompanied by a gradual and monotonous rise of the lateral displacement. For larger *t*, the energy of the skyrmion in the racetrack with Nd_2_Fe_14_B edges grows gently due to the slow displacement in the lateral direction. On the other hand, the energy of the skyrmion in the pure CoPt racetrack decreases drastically accompanied by a sharp increase of the lateral displacement *y*, where the skyrmion annihilates. One can see from the energy analysis that it is the large energy gradient at the racetrack edge due to high-*K* Nd_2_Fe_14_B that prevents the sharp lateral displacement and hence the annihilation of skyrmions, as can be seen clearly from Fig. 5(b). More detailed energy analysis are given in Figs S2 and S3.

**Figure 6:**
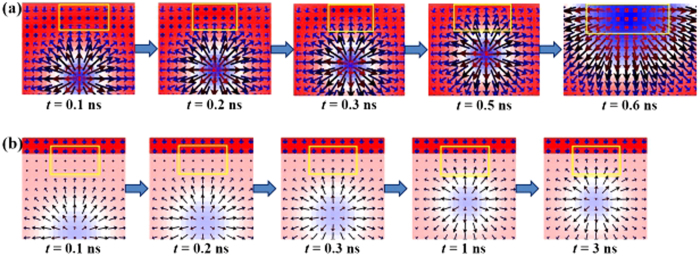
The spin distribution around the racetrack edge for (**a**) a pure 40 nm CoPt racetrack and (**b**) a CoPt racetrack rimed with 6 nm Nd_2_Fe_14_B edges. All the parameters used for the calculation are the same as the corresponding parameters adopted for the calculation in obtaining Fig. 5.

Why the high-*K* material can confine skyrmions within the racetrack can be seen more clearly by directly comparing the spin arrangements around the edge in both cases. The spin direction at the upper periphery of the skyrmion and that at the upper edge of pure CoPt racetrack are perpendicular when it is far away, as shown in Fig. 6(a) at *t* = 0.1 ns. As the skyrmion approaches the upper edge due to the Magnus force, the spin direction at the upper edge changes gradually towards that at the skyrmion upper periphery because of the exchange interaction, as shown in Fig. 6(a) for *t* = 0.2 ns, 0.3 ns and 0.5 ns. Finally the skyrmion is collapsed as shown in Fig. 6(a) at *t* = 0.6 ns. In contrast, the spin direction at the upper edge can be retained due to the high crystalline anisotropy of the edge material, as shown in Fig. 6(b) for a CoPt racetrack rimmed with Nd_2_Fe_14_B edges. Therefore, as the skyrmion approaches the upper edge due to the Magnus force, the exchange energy becomes larger and larger due to the huge difference between the nearby spins, which creates a large repulsion force so that the skyrmion is bounced back as shown in Fig. 2(c). Consequently, the annihilation of the skyrmion is avoided.

In summary, a new skyrmion-based racetrack memory has been designed by adding the high-*K* material at the racetrack edges, where skyrmions can move smoothly at a wide range of driving current. The new design solves both skyrmion clogging at the racetrack end and annihilation at the edge. Moreover, the driving current needed to achieve a high skyrmion speed is reduced in compared to previous works, demonstrating the possibility of building an ultrafast and energy-efficient skyrmion storage device in the future.

## Methods

### Micromagnetic simulations

The micromagnetic simulations are performed using the object-oriented micromagnetic framework (OOMMF)^37^ including the extension module of the Dzyaloshinskii-Moriya interaction (DMI)^38^,^39^,^40^ without temperature effect (*T* = 0). For simulations with spin-polarized current perpendicular to the racetrack plane (CPP)^2^, the time-dependent magnetization dynamics is governed by the modified Landau–Lifshitz–Gilbert (LLG) equation^41^,^42^,^43^,


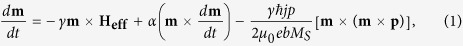


where **m** = **M/***M*_*s*_ is the reduced magnetization vector and *γ* is the Landau-Lifshitz gyromagnetic ratio. **H**_**eff**_ is the effective field and *α* is the Gilbert damping coefficient with the value between zero and one, while **p** is a spin polarization vector with the coefficient *p* and *b* is the thickness of the magnetic racetrack.

The effective field is a function of **M** defined as follows^2^,^41^,


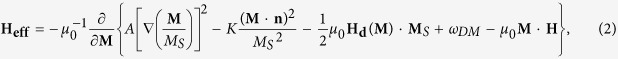


where *A* and *K* are the exchange and anisotropy energy constants, respectively. The five terms in the braces of Eq. (2) are Heisenberg exchange energy, the magnetic anisotropy energy, the demagnetization energy, the DMI energy and the Zeeman energy due to the external applied field.

The energy density of DMI in a continuous magnetization model is expressed as^2^,^43^


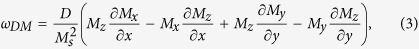


where *M*_*x*_, *M*_*y*_, *M*_*z*_ are the components of the magnetization **M** and *D* is the continuous effective DMI constant.

In the simulation, the middle part of the proposed racetrack is made of CoPt with a width of 40 nm, whilst the two symmetrical edge parts are made of a material with a higher anisotropy, *i.e.*, FePt, Nd_2_Fe_14_B or SmCo_5_ with width ranging from 1 nm to 6 nm. The material parameters are adopted from refs 2 and 44 shown in Table 3. A much smaller Dzyaloshinskii-Moriya interaction (DMI)^38^,^39^,^40^ value has been used in the present work for materials with a higher anisotropy to match the practical situation. A spin current with polarization of 0.4 is applied perpendicular to the racetrack plane (CPP), which results in much larger skyrmion velocities than with in-plane ones (CIP)^2^. Two typical damping constants^2^,^18^,^29^,^30^, *i.e.*, 0.1 and 0.3 are adopted to investigate the corresponding effect. For convenience, the average value of the exchange constant of CoPt and coating high-*K* materials is adopted for the interface exchange constant which yields the exchange constant between the CoPt and Nd_2_Fe_14_B as 11.35 pJ/m. The other interface exchange constants are 11.5 pJ/m and 13.5 pJ/m for the CoPt racetrack coated with FePt and SmCo_5_ respectively.

## Additional Information

**How to cite this article**: Lai, P. *et al*. An Improved Racetrack Structure for Transporting a Skyrmion. *Sci. Rep.* **7**, 45330; doi: 10.1038/srep45330 (2017).

**Publisher's note:** Springer Nature remains neutral with regard to jurisdictional claims in published maps and institutional affiliations.

## Supplementary Material

Supplementary Information

Supplementary Movie 1

Supplementary Movie 2

Supplementary Movie 3

## Figures and Tables

**Figure 1:**
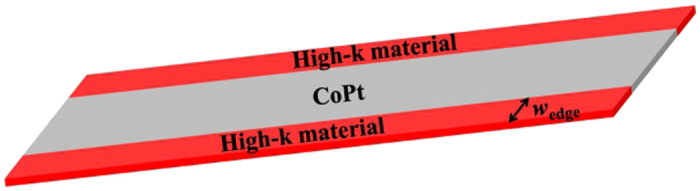
Schematic of the proposed skyrmion racetrack, where the middle part is made of CoPt, whilst the two symmetrical edge parts are made of a material with a higher anisotropy with a width denoted by *w*_*edge*_.

**Table 1:** Comparison of skyrmion velocity *v*_*x*_ and driving current *j* for different racetrack designed.

Racetrack designed	Way of current injection	*j* (MA/cm^2^)	p	*v*_*x*_ (m/s)	Refs
Rectangular CoPt racetrack	CPP	1	0.4	12	2,8,35
		5	0.4	46	18
		5	0.4	57	2, 8
Rectangular CoPt racetrack with a curb	CPP	10	0.7	130	29
	CIP	50	0.7	70	
		100	0.7	110	
CoPt racetrack with Nd_2_Fe_14_B edge	CPP	4–16	0.4	52–125	Fig. 4(a)
CoPt racetrack with SmCo_5_ edge	CPP	4–20	0.4	53–153	
CoPt racetrack with Nd_2_Fe_14_B edge	CPP	1–9	0.4	73–260	Fig. 4(b)
CoPt racetrack with SmCo_5_ edge	CPP	1–11	0.4	75–314	

**Table 2:** Comparison of parameters used in the present calculation, which are adopted from refs 2 and 29.

parameter	Figs 2, 3 and 4(a)	Fig. 4(b)
CoPt racetrack width *W* (nm)	40	60
Skyrmion diameter *D*_*S*_ (nm)	10	24
Magnetic crystalline anisotropy *K* (MJ/m^3^)	0.8	0.6
Spin polarization coefficient *P*	0.4	0.4
Gilbert damping constant *α*	0.3	0.1

For the CoPt racetrack edged with a high-*K* material, the width *W* denotes the inner CoPt racetrack width.

**Table 3:** Magnetic material parameters used in the present calculation, which are adopted from refs 2 and 44.

s parameters	material			
	CoPt	FePt	Nd_2_Fe_14_B	SmCo_5_
Saturation magnetization *M*_*s*_ (MA/m)	0.58	1.1	1.28	0.84
Exchange stiffness *A* (pJ/m)	15	8	7.7	12
Uniaxial anisotropy constant *K* (MJ/m^3^)	0.8	2.0	4.3	17.1
DMI constant *D* (mJ/m^2^)	3	0.1	0.1	0.1
