# Supplementary material for: An Improved Racetrack Structure for Transporting a Skyrmion
Source: Sci Rep. 2017 Mar 30;7:45330. doi: 10.1038/srep45330 (PMC5372177; doi:10.1038/srep45330)
Supplement: Supplementary Information [file srep45330-s1.pdf]

# An Improved Racetrack Structure for Transporting a Skyrmion

P. Lai[1, 2], G. P. Zhao[1, 3, *], H. Tang[1], N. Ran[1], S. Q. Wu[1], J. Xia[4], X. Zhang[4], Y. Zhou[4]

1. *College of Physics and Electronic Engineering, Sichuan Normal University, Chengdu 610101, China*
2. *Department of Physics and Electronic science, Aba Teachers University, Wenchuan 623002, China*
3. *Collaborative Innovation Center for Shanxi Advanced Permanent Materials and Technology, Linfen 041004, China*
4. *School of Science and Engineering, The Chinese University of Hong Kong, Shenzhen 518172, China*

*E-mail: zhaogp@uestc.edu.cn

## SUPPLEMENTERY INFORMATION

**Supplementary Movie 1.** Moving and annihilation of a skyrmion on the pure CoPt racetrack driven by a spin current of 8 MA/cm$^2$.

**Supplementary Movie 2.** Moving and clogging of a skyrmion on the CoPt racetrack rimmed with 6-nm-wide $Nd_2Fe_{14}B$ edges under a spin current of 8 MA/cm$^2$.

**Supplementary Movie 3.** Moving and passing of a skyrmion on the CoPt racetrack rimmed with 6-nm-wide $Nd_2Fe_{14}B$ edges under a spin current of 10 MA/cm$^2$.

### Comparison of skyrmion velocities for different edge DMI constants

Various DMI values have been used for the edge materials in the simulation which shows

that the skyrmion velocity and hence the annihilation of skyrmions are not sensitive to the DMI values of the edge materials. As shown in Fig. S1, the skyrmion velocity has negligible change as the DMI values for $Nd_2Fe_{14}B$ used in the simulation vary by three orders. In particular, in all the racetracks rimmed with $Nd_2Fe_{14}B$ at the edges, the skyrmion annihilation has been avoided. In contrast, the skyrmion annihilates at 0.6 ns in a pure CoPt racetrack, leading to the loss of the skyrmion signal.

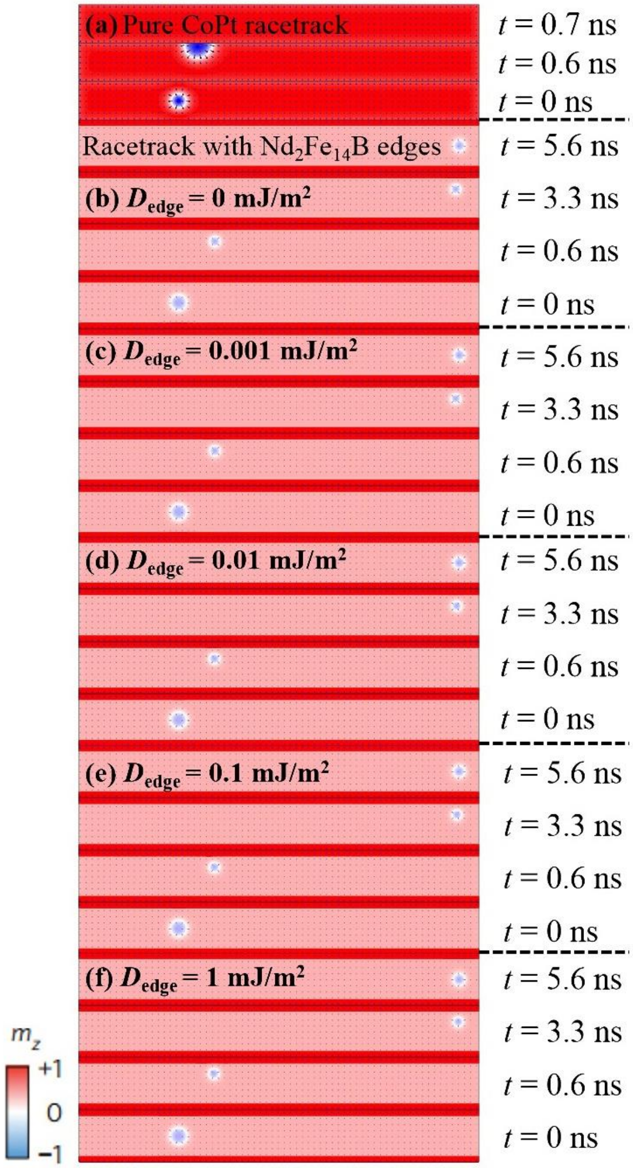

**Supplementary Figure S1.** Snapshots of simulation at various times $t$ for a skyrmion driven by the spin current of 8 $MA/cm^2$ in a pure CoPt racetrack and CoPt racetracks rimmed with 6-nm-wide $Nd_2Fe_{14}B$ edges. The DMI used in $Nd_2Fe_{14}B$ for (b), (c), (d), (e) and (f) are 0, 0.001, 0.01, 0.1 and 1 $mJ/m^2$, respectively.

**Time evolution of the skyrmion energy as well as the accompanied lateral and longitudinal displacement**

The total energy as functions of the simulation time for a skyrmion driven on the 40 nm CoPt racetrack with 6-nm-wide $Nd_2Fe_{14}B$ edges is shown in Fig. S2. The lateral and longitudinal displacements are also shown for comparison. The total energy increases fast for $t < 1$ ns, accompanied by an abrupt change of the lateral displacement, as shown in Fig. S2 (a). For 1 ns < $t$ < 3 ns, both the total energy and the lateral displacement are constants, which are $2.38 \times 10^{-21}$ J and 9.89 nm, respectively. For 3 ns < $t$ <3.5 ns, the total energy increases due to the interaction of the skyrmion with the right end, corresponding to a longitudinal displacement of 385 nm, as illustrated in Fig. S2 (b). For 3.5 ns < $t$ <4.2 ns, the total energy falls drastically due to the significant drop of the lateral displacement. For larger $t$, the total energy reaches a constant whilst the skyrmion locates at the right center of the racetrack.

In contrast, the total energy of a skyrmion driven on a pure CoPt racetrack falls drastically at $t$ =0.565 ns, accompanied by a significant increase of the lateral displacement as shown in Fig. S3 (a), indicating the annihilation of the skyrmion at the upper edge. More detailed description of

the skyrmion behavior near annihilation can be found in Fig. S3 (b).

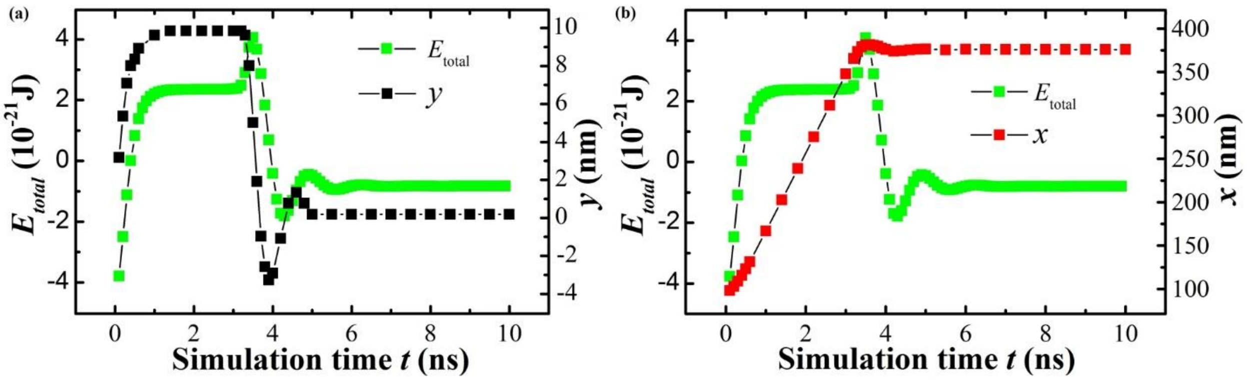


**Supplementary Figure S2.** Calculated total energy as functions of the simulation time for a skyrmion driven on the 40 nm CoPt racetrack with 6-nm-wide $Nd_2Fe_{14}B$ edges by a spin-current of 8 MA/cm$^2$. The lateral displacement *y* and longitudinal displacement *x* are shown in (a) and (b) respectively for comparison. The initial skyrmion energy ($t$ = 0) and the racetrack center are set as zero points for the total energy and lateral displacement, respectively.

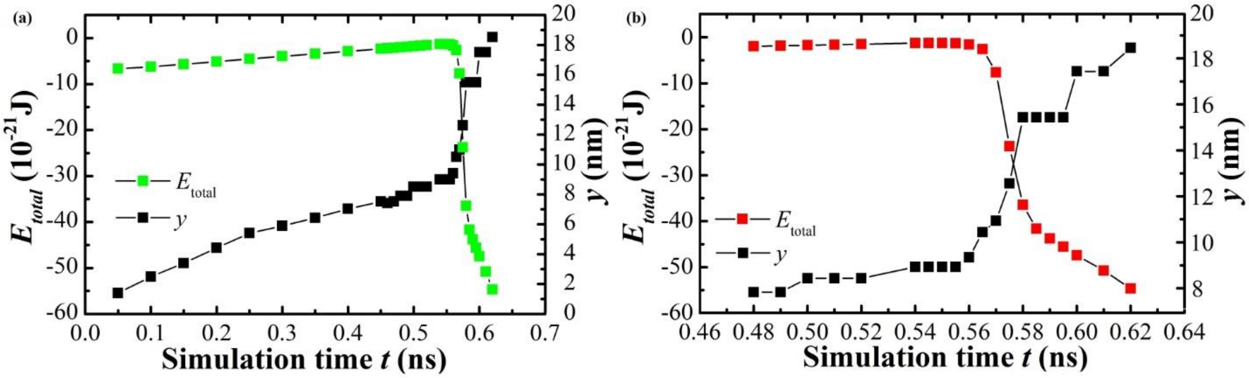


**Supplementary Figure S3.** Calculated total energy and lateral displacement *y* as functions of the simulation time for a skyrmion driven on the pure 40 nm CoPt racetrack by a spin-current of 8 MA/cm$^2$. The initial skyrmion energy ($t$ = 0) and the racetrack center are set as zero points for

the total energy and lateral displacement, respectively. A long ( $\Delta t$ =0.615 ns) and short ( $\Delta t$ =0.14 ns) simulation time span is selected for (a) and (b) respectively.
