# Supplementary figures and images for: An Improved Racetrack Structure for Transporting a Skyrmion
Source: Sci Rep. 2017 Mar 30;7:45330. doi: 10.1038/srep45330 (PMC5372177; doi:10.1038/srep45330)

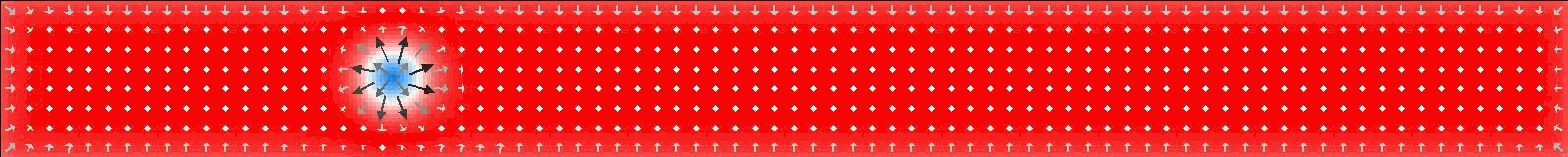

Supplement: Supplementary Movie 1 [file srep45330-s2.gif]

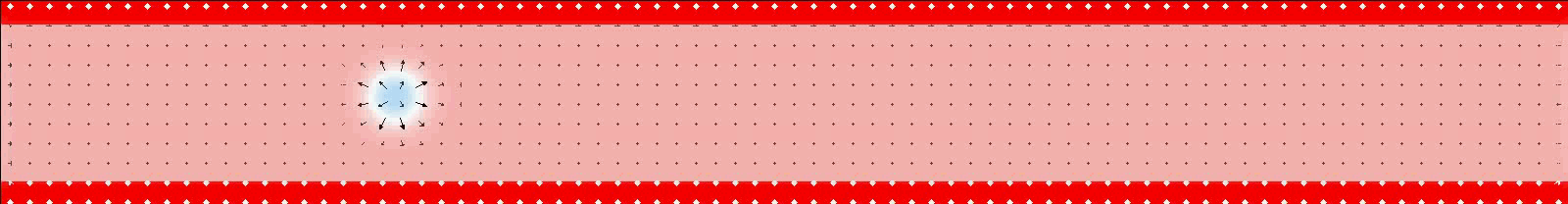

Supplement: Supplementary Movie 2 [file srep45330-s3.gif]

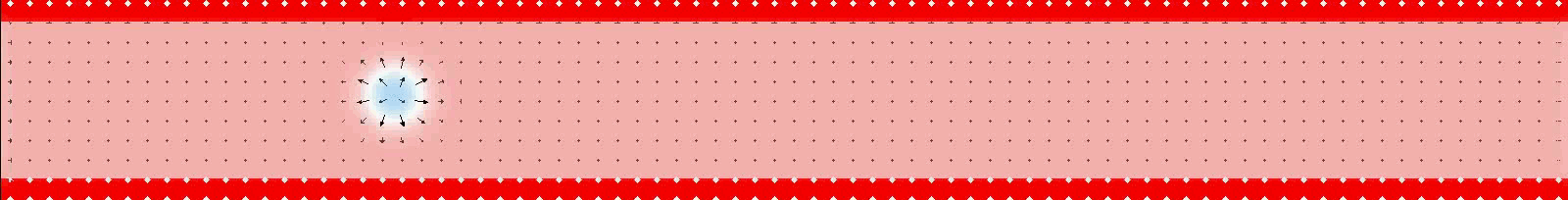

Supplement: Supplementary Movie 3 [file srep45330-s4.gif]
